# Beyond recommendations: expanding the ethical discourse on AI-assisted academic writing

**DOI:** 10.1186/s41077-025-00362-2

**Published:** 2025-06-02

**Authors:** Mahin Nosratzehi, Shahin Nosratzehi, Masoud Keikha

**Affiliations:** 1 Department of Internal Medicine, School of Medicine, Zahedan University of Medical Sciences, Zahedan, Iran; 2 Genetics of Non-Communicable Disease Research Center, Ali Ibne Abitaleb Hospital, Zahedan University of Medical Sciences, Zahedan, Iran; 3 Tropical and Communicable Diseases Research Center, Iranshahr University of Medical Sciences, Iranshahr, Iran; 4 Department of Medical Microbiology, School of Medicine, Iranshahr University of Medical Sciences, Iranshahr, Iran

## Abstract

In response to Cheng et al.’s article on ethical recommendations for artificial intelligence (AI)-assisted academic writing, we propose an expanded ethical discourse to address the evolving role of AI in scholarly communication. While applauding the authors’ foundational framework, we argue for greater disciplinary specificity, clearer thresholds for AI contribution, and broader consideration of systemic risks including linguistic bias, environmental impact, and corporate concentration. We advocate for the development of a graded typology of AI involvement, institution-led regulatory mechanisms, and integration of ethical AI use into editorial and research training practices. These enhancements are essential for building equitable, transparent, and sustainable AI governance in academic publishing.

## To the Editor,

We read with great interest the article by Cheng et al. (2025), “Artificial intelligence-assisted academic writing: recommendations for ethical use” [1]. The authors should be commended for initiating a timely and structured conversation on the use of artificial intelligence (AI) in scholarly writing. As AI technologies increasingly permeate research processes, the relevance of such guidance is indisputable. While the article provides a useful starting point, we believe a more expansive ethical discourse is needed. We wish to highlight areas that would benefit from further refinement and, importantly, propose constructive ways to build upon the current framework.

## Disciplinary variation and the limits of general guidelines

While Cheng et al. present broadly applicable recommendations, the ethical dimensions of AI use are far from uniform across academic fields. In disciplines such as computer science and quantitative biology, AI systems may form an integral part of experimental methodology, while in the humanities or qualitative social sciences, their role is typically confined to stylistic enhancement [2].

To promote relevance and compliance, we suggest that professional societies and journal editorial boards collaborate to develop discipline-specific ethical supplements, akin to field-tailored extensions of global research ethics codes. These could include examples of acceptable and unacceptable AI use cases in each field, benchmarks for disclosure, and practical checklists for authors. Moreover, a modular framework allowing journals to append their own discipline-relevant clauses to general AI policies could help contextualize ethical expectations.

## Clarifying the threshold of “substantial contribution”

The recommendation that AI tools should not qualify for authorship aligns with prevailing norms. However, ambiguity arises in defining what constitutes a “substantial contribution.” With current large language models capable of synthesizing literature, critiquing methodologies, and even generating hypotheses, the line between assistance and authorship is increasingly blurred [3].

We propose adopting a graded typology of AI involvement, ranging from.

- Type I: Mechanical assistance (e.g., grammar correction)
- Type II: Stylistic and linguistic enhancement
- Type III: Content suggestion or synthesis (e.g., summarizing literature)
- Type IV: Conceptual or analytical contribution (e.g., proposing frameworks or critiques)

Each level would carry corresponding disclosure obligations, with Type III and IV uses triggering editorial oversight or reviewer annotation. A similar matrix could be adopted by journals or incorporated into the CRediT taxonomy, enabling authors to classify AI input transparently.

## Broadening the ethical lens beyond plagiarism

The current emphasis on plagiarism and transparency overlooks broader, systemic ethical risks. Most generative AI models are trained on opaque, English-dominant corpora, potentially reproducing cultural biases and marginalizing non-Western knowledge systems [4]. Additionally, the environmental cost of AI training and the concentration of model ownership within a few corporations raise concerns about sustainability and knowledge monopolies [5].

We urge that future ethical frameworks explicitly address these dimensions. For example:

- Linguistic equity could be promoted through the development and citation of regionally fine-tuned open-source models.
- Environmental responsibility could be integrated into AI ethics through reporting of carbon footprints for large-scale deployments, following emerging norms in green computing.
- Model provenance disclosures could be required, prompting authors to specify the training origins and limitations of the AI tools they employed.

These steps would foster a more globally just and environmentally sustainable research ecosystem.

## Institutional responsibility and regulatory frameworks

While the authors rightly stress individual transparency, ethical stewardship should extend beyond personal responsibility. Editorial boards, research institutions, and publishers must play an active role in governing AI use.

We recommend the following institutional initiatives:

- Mandatory AI-use statements standardized across journals, modeled after existing conflict-of-interest disclosures
- Reviewer training modules to sensitize peer reviewers to the ethical and technical nuances of AI-generated content
- Integration of AI ethics into research integrity curricula, ensuring that early-career researchers receive foundational training in this evolving domain
- National and international academic bodies (e.g., COPE, ICMJE) should collaborate to formulate regulatory frameworks on permissible AI involvement across the publication lifecycle

Such multilevel governance can ensure that ethical AI use is not only encouraged but systematically reinforced.

## Conclusion

Cheng et al. have initiated an essential conversation. We echo their call for ethical AI use in academic writing but believe that more nuanced, specific, and action-oriented approaches are required. The future of scholarship in an AI-mediated world depends on frameworks that evolve with technological capability while respecting disciplinary variation, epistemic justice, and environmental responsibility. By offering concrete proposals from typologies of AI involvement to environmental accountability we hope our contribution encourages further dialogue and supports the development of more robust ethical stewardship.

## Data Availability

No datasets were generated or analysed during the current study.
